# The Invasive Wetland Plant *Alternanthera philoxeroides* Shows a Higher Tolerance to Waterlogging than Its Native Congener *Alternanthera sessilis*

**DOI:** 10.1371/journal.pone.0081456

**Published:** 2013-11-26

**Authors:** Yue Chen, Ya Zhou, Tan-Feng Yin, Chun-Xiang Liu, Fang-Li Luo

**Affiliations:** School of Nature Conservation, Beijing Forestry University, Beijing, China; Key Laboratory of Horticultural Plant Biology (MOE), China

## Abstract

Plant invasion is one of the major threats to natural ecosystems. Phenotypic plasticity is considered to be important for promoting plant invasiveness. High tolerance of stress can also increase survival of invasive plants in adverse habitats. Limited growth and conservation of carbohydrate are considered to increase tolerance of flooding in plants. However, few studies have examined whether invasive species shows a higher phenotypic plasticity in response to waterlogging or a higher tolerance of waterlogging (lower plasticity) than native species. We conducted a greenhouse experiment to compare the growth and morphological and physiological responses to waterlogging of the invasive, clonal, wetland species *Alternanthera philoxeroides* with those of its co-occurring, native, congeneric, clonal species *Alternanthera sessilis*. Plants of *A. philoxeroides* and *A. sessilis* were subjected to three treatments (control, 0 and 60 cm waterlogging). Both *A. philoxeroides* and *A. sessilis* survived all treatments. Overall growth was lower in *A. philoxeroides* than in *A. sessilis*, but waterlogging negatively affected the growth of *A. philoxeroides* less strongly than that of *A. sessilis*. *Alternanthera philoxeroides* thus showed less sensitivity of growth traits (lower plasticity) and higher waterlogging tolerance. Moreover, the photosynthetic capacity of *A. philoxeroides* was higher than that of *A. sessilis* during waterlogging. *Alternanthera philoxeroides* also had higher total non-structural and non-soluble carbohydrate concentrations than *A. sessilis* at the end of treatments. Our results suggest that higher tolerance to waterlogging and higher photosynthetic capacity may partly explain the invasion success of *A. philoxeroides* in wetlands.

## Introduction

Plant invasion is a worldwide problem and considered to pose as an environmental and economic threat to human well-being [1]–[5]. A commonly used approach to study plant invasiveness is to compare the traits of invasive and non-invasive plant species [5]–[8], but results have not been consistent [9]–[12]. Various studies have found invasive species have a higher growth rate [11], resource use efficiency [13], reproductive capacity [14] or fecundity [11] than non-invasive species. In a study comparing 20 ecologically and phylogenetically related species pairs, invasive species were more capable of carbon acclimation and had higher performance under limited resource availabilities than non-invasive species [15]. In a meta-analytic study, invasive plants showed significantly higher values than non-invasive species in six trait categories, including physiology, leaf-area allocation, shoot allocation, growth rate, size and fitness [12]. However, other studies have found that invasive species did not have higher growth rate or resource use efficiency than non-invasive species [2], [9]–[10].

Phenotypic plasticity is thought to be important for plants as it allows them to grow in a wide range of environments [8]–[10], [16], but there are different views on the relationship between phenotypic plasticity and plant invasion [2], [5], [10]. In some studies, invasive plants were found to possess higher plasticity than non-invasive species, and higher phenotypic plasticity was considered to facilitate invasion [2], [8]-[9], [12]. In other studies, invasive plants showed lower plasticity than non-invasive plants [15], [17], or showed similar plasticity but higher tolerance to changing light and nutrient availability [9]–[10], [15]. Therefore, plant invasiveness may be determined, not only by phenotypic plasticity, but also by higher values of some key traits, such as those related to physiology, biomass allocation, growth rate, size and fitness [7], [12], [15], [18].

For some introduced plants, a higher tolerance to stressful conditions was found to facilitate invasion in adverse habitats [6], [16], [19]–[21]. Waterlogging is a common stress for wetland plants [22]. The major impacts of waterlogging are inhibition of photosynthetic capacity, restriction of energy and carbohydrate availability, and reduction of growth and developmental processes in many riparian plants due to slow rates of gas diffusion and severe shading [22]–[23]. Compared to waterlogging-sensitive plants, tolerant plants can better maintain photosynthetic capacity and conserve energy and carbohydrate by restricted growth performance, and this could positively affect the survival rate and generation of new tissues after re-emergence following flooding [23]–[24]. Recently, the ability to tolerate waterlogging was found to help explain the invasion of *Spartina alterniflora* in coastal wetlands in China [25]. However, it is still not clear whether invasive plants in wetlands have higher values of traits related to photosynthetic capacity, carbohydrate metabolism and growth processes than non-invasive plants.

In this study, we subjected a wetland invader *Alternanthera philoxeroides* (Martius) Grisebach (Amaranthaceae; alligator weed) and its co-occurring, native, congener *Alternanthera sessilis* (L.) DC. (sessile joyweed) to a control (no waterlogging, normal daily watering), 0-cm-deep waterlogging (soil saturated with water) and 60-cm-deep waterlogging, and compared their responses. We aimed to test (1) whether the invasive species *A. philoxeroides* shows a higher phenotypic plasticity or higher tolerance in response to waterlogging (lower plasticity) than its native congener *A. sessilis*, and (2) whether the invasive species shows a higher growth rate, photosynthetic capacity and carbohydrate accumulation than its native congener.

## Materials and Methods

### Plant species

*Alternanthera philoxeroides* is a perennial herb, and can grow in aquatic, semi-aquatic and terrestrial environments [26]–[27]. It originates from South America, and is listed as a highly invasive species in many countries, including China. This species can spread quickly by vegetative propagation, and produces hollow, creeping stolons which may turn upright at the end. Its leaves are opposite and orbicular to ovate. In China, *A. philoxeroides* blooms from May to November, but rarely produces viable seeds. This species is now widespread in South China, and causes great ecological and economical problems.

*Alternanthera sessilis* is a perennial herb and the only native congener of *A. philoxeroides* in China [28]–[29]. It can also spread by clonal growth. Although both species have similar morphology and can co-occur in wetlands, *A. philoxeroides* shows a stronger adaptability to different water availabilities and often outperforms *A. sessilis* in extremely diverse habitats from swamps to dry lands [28]–[29].

### Material preparations and experimental design

In May 2011, plants of each species were collected from at least five locations at least 10 m apart in each of two wetlands in Taizhou, Zhejiang province, China. The sampling sites are two derelict wetlands which do not belong to the parts of any farms or national parks; thereby we did not need any relevant permits for collecting plant samples. Our studies did not involve endangered or protected species. Plants from the different locations were mixed and vegetatively cultivated for two months in a greenhouse of the Forestry Science Company of Beijing Forestry University. For each species, 75 stolon fragments, each having three nodes and an apex, were cut off from the stock population and put into tap water to facilitate rooting. We then selected 60 stolon fragments (ca. 7.5 cm long) and planted them in pots (17 cm diameter ×14 cm height) filled with a 1∶1 (v∶v) mixture of peat and sand containing 0.48 g kg^−1^ total nitrogen, 0.65 g kg^−1^ total phosphorus and 7.83 g kg^−1^ total organic carbon.

After one week of recovery, 56 plants of similar size of each species were selected for the experiment. Eight of the plants of each species were randomly selected and dried to measure initial biomass (*A. sessilis*: 0.91 ± 0.14 g, mean ± SE; *A. philoxeroides*: 0.96 ± 0.11 g). Sixteen of the remaining 48 plants were randomly assigned to each of three treatments, i.e. control (no waterlogging, i.e. normal daily watering), 0 cm waterlogging (water level kept at the soil surface level) and 60 cm waterlogging (water level 60 cm above the soil surface). The waterlogging treatments were conducted in twelve plastic boxes (90×70×70 cm, with four pots of each species per box, and four boxes per treatment). For the 0 and 60 cm waterlogging treatments, tap water was added to the boxes to compensate for water evaporation during the treatments.

The experiment lasted two months, from 16 July to 16 September 2011, and was conducted in the greenhouse. Eight plants in each treatment and species were randomly selected from four boxes (two plants per box) to be harvested on day 30 (i.e. 30 days after the beginning of the experiment); and the other eight were harvested on day 60 (60 days after the beginning of the experiment, i.e. the end of the experiment). During the experiment, the plants were watered daily; the air temperature ranged from 27 to 36°C; and the air relative humidity was around 60%. All plants survived until harvest.

### Measurements of growth and morphology

At each harvest date, we counted the numbers of stolons and leaves, and measured total stolon length and internode length of each plant. Each plant was then separated into stolons, leaves and roots, dried at 60°C for 48 h, and weighed.

### Measurements of photosynthetic capacity

At each harvest date, five plants from each treatment and species were randomly selected and used for measuring photosynthetic capacity. The net photosynthetic rate (Pn) of the selected plants was measured at 9:00–12:00 h on the youngest fully expanded leaf using a Li-6400 portable photosynthesis system (Li-Cor Biosciences, Lincoln, NE, USA) at a CO_2_ concentration of 400 µmol mol^−1^ and a photo flux density of 800 µmol m^−2^ s^−1^. The leaf area used for the measurements was obtained by scanning.

Fluorescence parameters were measured on the leaves opposite to those used for measuring Pn using a portable modulated fluorometer (PAM-2500, Heinz Walz, Germany) on the same day at 9:00–12:00 h. The minimum and maximum fluorescence in dark-adapted leaves (Fo and Fm) were measured after 30 minutes of dark adaptation by using leaf clips. Fo was measured using modulated light that was sufficiently low (<0.1 µmol m^−2^ s^−1^). Fm was measured using a 0.8 s saturating pulse at 8000 µmol m^−2^ s^−1^. The maximum quantum efficiency of PSII was calculated as Fv/Fm = (Fm−Fo)/Fm. The leaves were then exposed to white actinic light at an intensity of 800 µmol m^−2^ s^−1^ for 4 min. The same process was conducted to obtain the steady-state value of fluorescence (Fs) and the maximal light-adapted fluorescence level (Fm′). The effective quantum yield of PSII in the light were calculated as Yield = (Fm′−Fs)/Fm′, and the electron transport rate as ETR = ΔF/Fm′×0.5×PAR×0.84 [30].

### Chlorophyll determinations

Concentrations of chlorophyll *a* and *b* were measured on the same leaves used for measuring Pn. Leaf discs (0.89 cm^2^) were sampled from the middle of the lamina using a hole puncher immediately after the photosynthesis measurements. The concentrations of chlorophyll *a* and *b* in leaves were determined [31] with a UV-2550 spectrophotometer (Shimadzu Co, Kyoto, Japan).

### Measurements of non-structural carbohydrates

Four replicate plants per treatment and species were randomly selected for measurement of non-structural carbohydrates. Dried roots, stolons and leaves of the selected plants were separately ground into powder. About 15 mg of the powder from each plant part was used for analysis. Soluble sugars and non-soluble sugars were analyzed using the perchloric acid/anthrone method [32]. This method has been proven to be robust and frequently used to analyze carbohydrates in storage organs [33]. The soluble sugars were extracted from dried material with 0.5 ml of 80% ethanol at 80°C for 30 min. The extract was then centrifuged at 16000 r for 3 min, and the supernatant was collected. This process was repeated twice, 1.5 ml of 80% ethanol was used at the last time. Ethanol (80%) was added to the supernatant to increase the volume to 2 ml, and the supernatant was mixed thoroughly. Then, 30 µl of the supernatant was mixed with 70 µl water, 0.15 ml anthrone reagent (1 g anthrone ethyl dissolved in 50 ml ethyl acetate) and 1.5 ml concentrated sulfuric acid, and heated at 100°C for 15 min. The concentration of soluble sugars was determined by measuring the absorbance at 630 nm in a spectrophotometer, and subtracting the absorbance value of blank samples. These values were then regressed against readings from a set of standard solutions of glucose. The non-soluble sugars were extracted after hydrolyzing the residue with 1 ml of perchloric acid (35%) for 2 h. The extract was then centrifuged at 16000 r for 3 min, and the supernatant was used for determinations. The solubilised carbohydrate was analyzed using the anthrone reaction with the method previously described for soluble sugars. In this case, the readings were regressed against another set of standard glucose solutions. Results were expressed as the percentage (w/w) of sugars per unit of dry matter. The concentration of total non-structural carbohydrates was calculated as the sum of the concentrations of soluble and non-soluble sugars.

### Data analysis

Two-way ANOVAs were used to test the effects of species (*A. sessilis vs. A. philoxeroides*) and treatments (control, 0 and 60 cm waterlogging) on growth (biomass, total stolon length, number of stolons and leaves), morphology (the longest internode length and root to shoot ratio), photosynthetic parameters (Pn, Fv/Fm, Yield and ETR), and non-structural carbohydrate and chlorophyll concentrations on day 30 and 60 separately. SPSS 17.0 (SPSS, Chicago, IL, USA) was used for all analyses. Results were considered to be significantly if *P*<0.05.

## Results

### Growth traits

**Figure 1:**
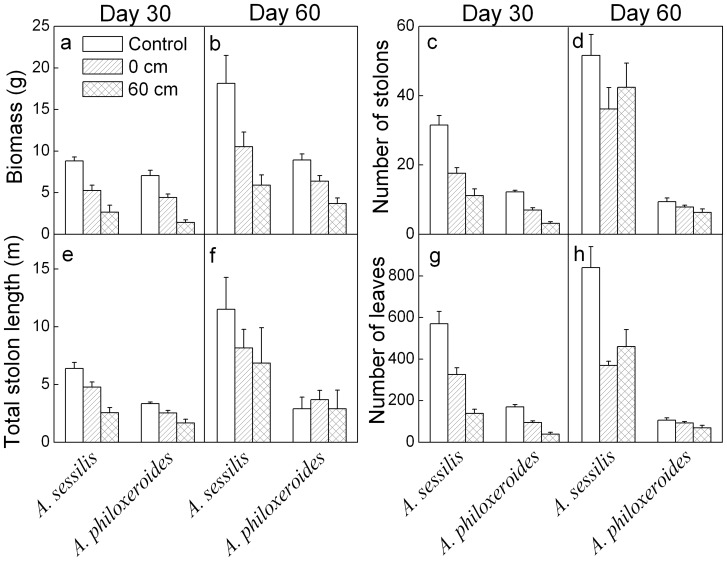
Mean values (+SE, n = 8) for growth traits of *Alternanthera sessilis* and *Alternanthera philoxeroides* subjected to different levels of waterlogging on day 30 and 60.

On both day 30 and 60, all growth measures (biomass, number of stolons, total stolon length and number of leaves) were lower in the invasive *A. philoxeroides* than in its native congener *A. sessilis* (Table 1a, Figure 1). Generally, the growth of both *A. philoxeroides* and *A. sessilis* decreased with increasing waterlogging intensity, except for number of stolons which was not affected by waterlogging on day 60 (Table 1a, Figure 1). However, the effects of waterlogging on the growth of the two species differed greatly (Table 1a, significant interaction effects). On day 30, waterlogging decreased number of stolons, total stolon length and number of leaves less in *A. philoxeroides* than in *A. sessilis* (Table 1a, Figure 1). On day 60, waterlogging significantly decreased total stolon length and number of leaves in *A. sessilis* but not in *A. philoxeroides* (Table 1a, Figure 1f and h).

**Table 1:** Effects of species (*Alternanthera sessilis vs. Alternanthera philoxeroides*), treatments (control, 0 cm and 60 cm waterlogging) and their interaction on the growth, morphological and physiological traits on day 30 and 60, respectively.

	Day 30			Day 60		
	Species	Treatment	S×T	Species	Treatment	S×T
(a) Growth trait						
Biomass	7.3^*^	52.1^***^	0.3^ns^	8.6^**^	9.2^**^	1.5^ns^
Number of stolons	96.0^***^	44.9^***^	7.0^**^	83.3^***^	1.7^ns^	1.1^ns^
Total stolon length	46.2^***^	28.0^***^	4.4^*^	89.9^***^	5.1^*^	6.0^**^
Number of leaves	100.3^***^	45.1^***^	12.8^***^	114.1^***^	12.0^***^	9.9^***^
(b) Morphological trait						
Root to shoot ratio	28.4^***^	6.3^**^	2.9^#^	100.9^***^	34.4^***^	17.8^***^
Longest internode length	<0.1^ns^	37.6^***^	0.5^ns^	4.9^*^	4.4^*^	<0.1^ns^
(c) Physiological trait						
Net photosynthetic rate(Pn)	33.3^***^	0.5^ns^	0.2^ns^	34.9^***^	9.7^**^	3.4^*^
Maximal efficiency of PSII (F_v_/F_m_)	7.0^*^	3.2^#^	4.8^*^	1.1^ns^	4.7^*^	0.3^ns^
Effective quantum yield of PSII (Yield)	0.2^ns^	15.8^***^	0.3^ns^	1.8^ns^	12.5^***^	2.8^ns^
Electron transport rate (ETR)	0.2^ns^	15.7^***^	0.3^ns^	1.8^ns^	12.6^***^	2.8^ns^
Total non-structural carbohydrate concentration	1.3^ns^	0.2^ns^	0.3^ns^	12.6^**^	5.4^*^	0.6^ns^
Soluble sugar concentration	1.2^ns^	0.3^ns^	2.3^ns^	2.5^ns^	3.3^ns^	2.5^ns^
Non-soluble sugar concentration	0.0^ns^	0.4^ns^	3.3^ns^	25.4^***^	3.2^ns^	2.5^ns^
Concentration of chlorophyll *a* and *b* (Ct)	6.7^*^	10.0^**^	0.3^ns^	3.6^#^	5.2^*^	3.5^#^
Concentration of chlorophyll *a* (Ca)	9.0^*^	9.7^ **^	0.5^ns^	3.4^#^	5.4^ *^	3.4^#^
Concentration of chlorophyll *b* (Cb)	1.2^ns^	10.3^**^	0.1^ns^	3.9^#^	4.0^ *^	3.3^#^

Values are *F*. Symbols show *p* (^***^ *p*<0.001, ^***^ *p*<0.01, ^*^ *p*<0.05, ^#^ *p*<0.1 and ^ns^ *p*≥0.1)). Degrees of freedom for the effects of species, treatment and their interaction are respectively (1, 47), (2, 47) and (2, 47) for growth and morphological traits, and (1, 29), (2, 29) and (2, 29) for physiological traits except soluble and non-soluble sugar concentrations, for which degree of freedoms are (1, 23), (2, 23) and (2, 23).

### Morphological traits

**Figure 2:**
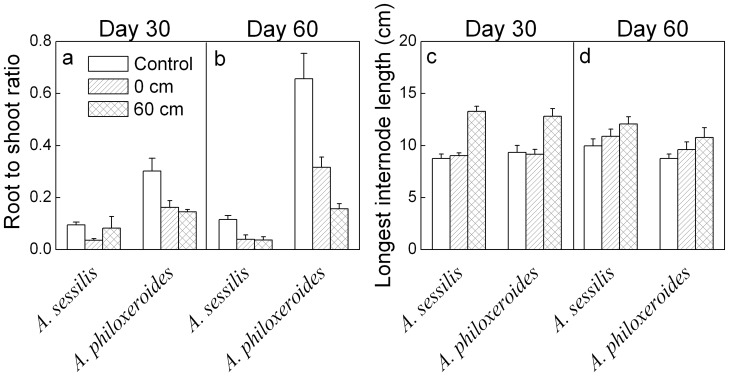
Mean values (+SE, n = 8) for morphological traits of *Alternanthera sessilis* and *Alternanthera philoxeroides* subjected to different levels of waterlogging on day 30 and 60.

On both day 30 and 60, root to shoot ratio was significantly lower in *A. sesslis* than in *A. philoxeroides* (Table 1b, Figure 2a and b). Waterlogging generally decreased root to shoot ratio of both species on both days, but more strongly in *A. philoxeroides* than in *A. sessilis* on day 60 (Table 1b, Figure 2b). Length of the longest stolon internode did not differ significantly between the two species on day 30, but it was significantly larger in *A. sesslis* than in *A. philoxeroides* on day 60 (Table 1b, Figure 2c and d). Waterlogging increased the length of longest internode in both species on both day 30 and 60 (Table 1b, Figure 2c and d).

### Physiological traits

**Figure 3:**
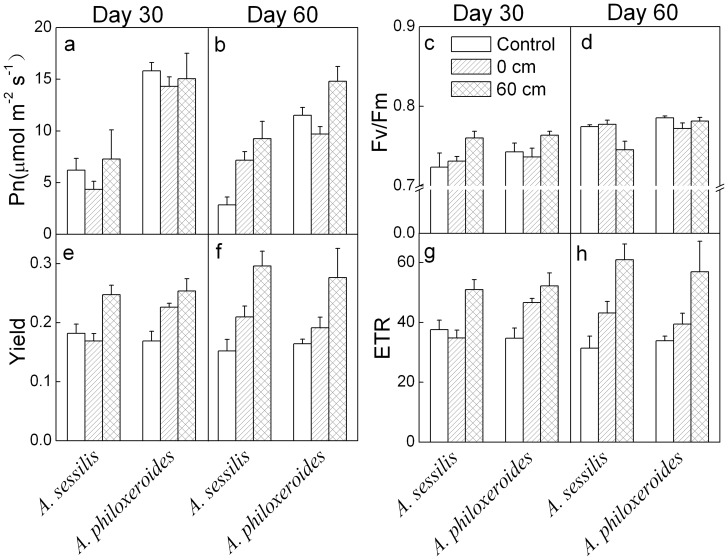
Mean values (+SE, n = 5) for photosynthesis parameters of *Alternanthera sessilis* and *Alternanthera philoxeroides* subjected to different levels of waterlogging on day 30 and 60. Fv/Fm - maximal efficiency of PSII photochemistry, Pn - net photosynthetic rate, Yield - fluorescent quantum yield, and ETR - electron transport rate.

On both day 30 and 60, the net photosynthetic rate (Pn) was lower in *A. sessilis* than in *A. philoxeroides* (Table 1c, Figure 3a and b). On day 30, waterlogging did not affect Pn of either species; on day 60, it increased Pn in both species and the effect was larger in *A. sessilis* than in *A. philoxeroides* (Table 1c, Figure 3a and b). The maximum quantum efficiency of PSII (Fv/Fm) was significantly lower in *A. sessilis* than in *A. philoxeroides* on day 30, but did not differ significantly between the two species on day 60 (Table 1c, Figure 3c and d). Waterlogging significantly affected Fv/Fm on day 60 and marginally (*P*<0.1) affected Fv/Fm on day 30 (Table 1c, Figure 3c and d). On day 30, 60 cm waterlogging increased Fv/Fm more greatly in *A. sessilis* than in *A. philoxeroides* (Table 1c, Figure 3c). On day 60, 60 cm waterlogging significantly decreased Fv/Fm in *A. sessilis*, but not in *A. philoxeroides* (Table 1c, Figure 3d). Neither effective quantum yield (Yield) nor electron transport rate (ETR) differed significantly between the two species. Waterlogging, especially the 60 cm waterlogging treatment, markedly increased Yield and ETR in both species on both day 30 and 60 (Table 1c, Figure 3e-h).

**Figure 4:**
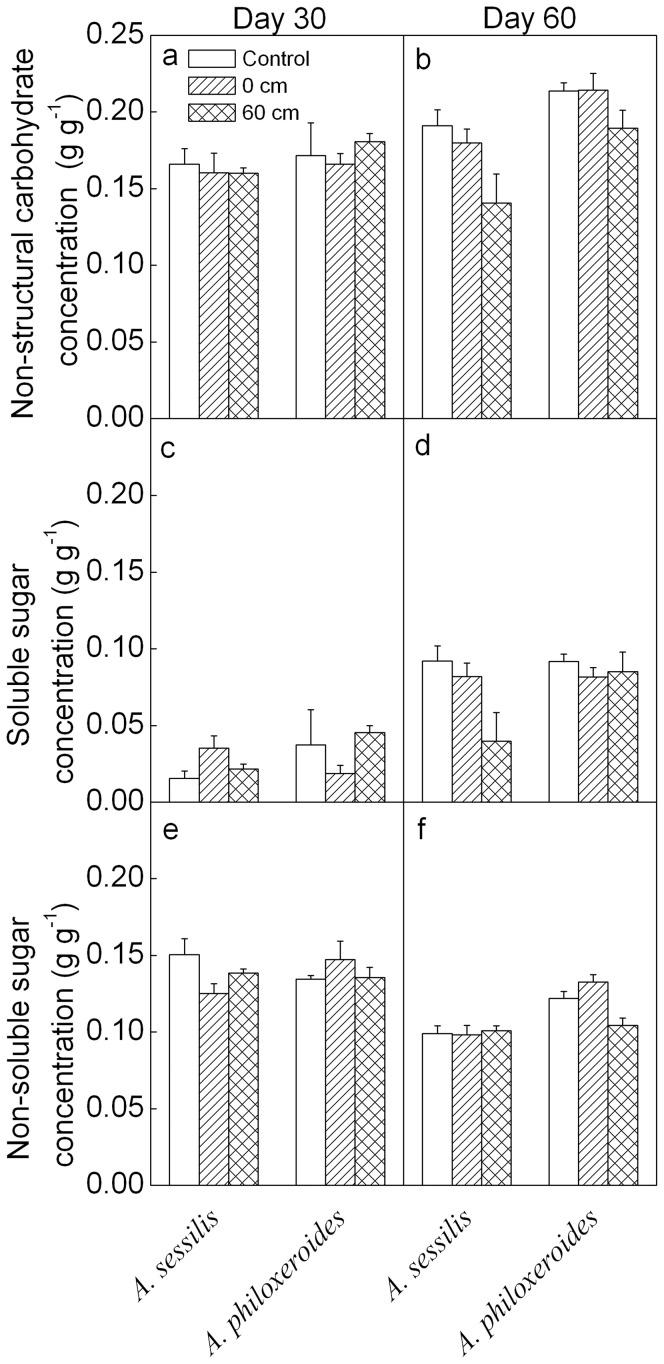
Mean values (+SE, n = 4) for carbohydrate concentrations of *Alternanthera sessilis* and *Alternanthera philoxeroides* subjected to different levels of waterlogging on day 30 and 60.

On day 30, the concentrations of total non-structural carbohydrate, soluble sugar or non-soluble sugar did not differ significantly between the two species (Table 1c; Figure 4). On day 60, the concentrations of total non-structural carbohydrate and non-soluble sugar were lower in *A. sessilis* than in *A. philoxeroides* (Table 1c, Figure 4b and f), and waterlogging significantly decreased non-structural carbohydrate concentration (Figure 4b).

**Figure 5:**
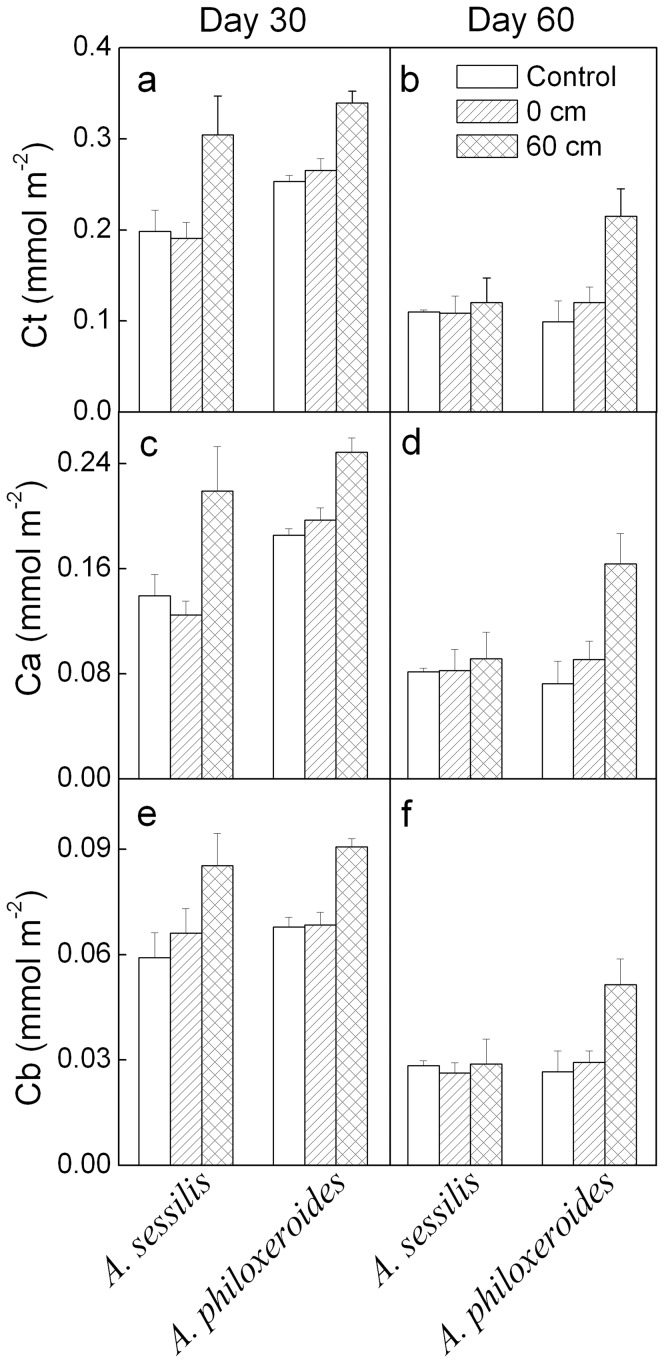
Mean values (+SE, n = 5) for chlorophyll concentrations of *Alternanthera sessilis* and *Alternanthera philoxeroides* subjected to different levels of waterlogging on day 30 and 60. Ct - concentration of chlorophyll *a* and *b*, Ca - concentration of chlorophyll *a*, and Cb - concentration of chlorophyll *b*.

On day 30, the concentrations of total chlorophyll (Ct) and chlorophyll *a* (Ca) were lower in *A. sessilis* than in *A. philoxeroides* (Table 1c, Figure 5c and e). Waterlogging, especially the 60 cm waterlogging treatment, significantly increased Ct, Ca and chlorophyll *b* (Cb) on both day 30 and 60 (Table 1c, Figure 5), and the effects were marginally larger in *A. philoxeroides* than in *A. sessilis* on day 60 (Table 1c, Figure 5, interaction effects, *P*<0.1)

## Discussion

Waterlogging decreased the growth of both the introduced, invasive species *A. philoxeroides* and the native species *A. sessilis*. However, waterlogging decreased the growth of these two species much less than that of flood-sensitive species [34]–[36], and even the 60 cm waterlogging treatment did not result in the death of any of the plants. Therefore, both species have a high capacity to tolerate waterlogging.

### Differences in tolerance to waterlogging

Waterlogging enhanced stolon elongation in both *A. philoxeroides* and *A. sessilis* (Table 1b, Figure 2c and d), indicating an escape strategy, which was significantly stronger in *A. sesslis* than in *A. philoxeroides* at the end of the experiment. Shoot elongation depends largely on cell elongation which requires synthesis of new cell walls, and hence the availability of energy and carbohydrates [37]. Thus, the stronger shoot elongation might lead to faster carbohydrate depletion [23]–[24].

It is commonly considered that phenotypic plasticity plays an important role in the invasiveness of introduced plants [8]–[10]. *Alternanthera philoxeroides* showed greater phenotypic and physiological plasticity than *A. sessilis* when subjected to heat or drought stress [28]–[29]. However, in the present study, the growth traits of *A. philoxeroides* were decreased less than those of *A. sessilis* under waterlogging conditions, indicating lower plasticity of growth traits (Table 1, Figures 1, 2 and 4). Previous studies generally showed that the negative impacts are greater in flood-sensitive plants than in flood-tolerant plants [23], [34], [38]. The flood-tolerant plants are often characterized by restricted growth performance and conservation of energy and carbohydrates, especially during complete flooding [24]. Our results are consistent with these findings and suggest that *A. philoxeroides* is less sensitive to waterlogged conditions than *A. sessilis*. On the other hand, *A. philoxeroides* showed a higher root to shoot ratio and chlorophyll concentrations (Table 1b and c, Figures 2 and 5), which might improve light interception, O_2_ availability and carbohydrate status of plants, and reduce respiratory loss [22], [35], [39]. Furthermore, we observed that *A. philoxeroides* lost more than 2/3 of its submerged leaves (Y. Chen, pers. obs.), which might be a strategy to reduce respiration [35], [40]. Relatively low sensitivity of growth traits, high values of root to shoot ratio and chlorophyll concentrations and loss of underwater leaves might contribute to the tolerance of *A. philoxeroides* to waterlogged conditions.

Compared to *A. philoxeroides*, *A. sessilis* showed a relatively stronger decrease in growth traits, suggesting higher sensitivity to waterlogging (Figure 1). Unlike *A. philoxeroides*, *A. sessilis* retained most of its underwater leaves during the experiment (Figure 1; Y. Chen, pers. obs.). The greater shoot biomass of *A. sessilis* might allow higher underwater photoassimilation [22], [39], but also cause higher respiratory rate and lead to lower carbohydrate accumulation [35], [40]. Thus, higher sensitivity of growth traits and higher carbohydrate consumption might decrease the tolerance of *A. sessilis* to prolonged, deep submergence.

### Differences in growth and physiological traits

*Alternanthera philoxeroides* and *A. sessilis* differed in growth and physiological traits. *Alternanthera philoxeroides* generally displayed greater ability to maintain chlorophyll and photosynthesis to accumulate carbohydrates than *A. sessilis* (Table 1, Figures 3–5). These results agree with previous findings that invasive species can have significantly higher values of some physiological traits than native species, including traits for photosynthesis, transpiration, nitrogen content, nitrogen use efficiency and water use efficiency [12], [15], [18]. Similarly, many studies have shown that invasive species have significantly higher growth rate than non-invasive species [11]–[12]. However, we found that the values of all growth traits of *A. philoxeroides* were lower than that of *A. sessilis* (Table 1a, Figure 1). Other previous work has similarly found that invasive species did not consistently outperform co-occurring native species in terms of specific leaf area, growth rate, competitive ability, or fecundity, and that differences largely depend on environmental conditions, including resource levels and disturbance regimes [2], [6], [20], [41]–[42]. Besides, larger size leads to decreased stress tolerance in *Centaurea stoebe* [43]. In *A. philoxeroides*, the small size might confer high tolerance.

The inconsistency between lower values of growth traits and higher values of physiological traits in *A. philoxeroides* than in *A. sessilis* might be caused by leaf shedding and higher partitioning of biomass to roots (Figure 2; Y. Chen, pers. obs.). Lower biomass and leaf size in *A. philoxeroides* than in *A. sessilis* are also found during the switch from wet conditions to drought [28]. Relatively low biomass allocation to shoots and leaf shedding, but relatively high carbon asssimilation in emerged leaves might lead to a higher carbohydrate accumulation in *A. philoxeroides* than in *A. sessilis* (Figures 1 and 4; Y. Chen, pers. obs.).

### Performance in waterlogged and terrestrial conditions

*Alternanthera philoxeroides* can show a much higher competitive ability than *A. sessilis* in both waterlogged and drier conditions [28]. The broad ecological niche of *A. philoxeroides* contrasts with the ecological ranges of some other invasive wetland species, such as *Phragmites australis*, *Eichhornia crassipes* and species of *Spartina*, which can only invade the aquatic environment [25], [42], [44]–[45]. When the depth of floodwater increased, *A. philoxeroides* allocated less biomass to roots and produced longer internodes (Table 1b, Figure 2). The pattern is completely reversed with decreasing water availability [28]. Plants preferentially grow vegetative organs to maximize the surface for uptake of the most limiting resources [46]. In waterlogged conditions, *A. philoxeroides* increased shoot biomass allocation, which could increase O_2_, CO_2_ and light uptake, whereas plants in drier conditions have a greater belowground biomass, which can increase water uptake. The ability of leaves of *A. philoxeroides* to maintain photosynthetic capacity in waterlogged conditions could allow rapid carbon gain once leaves re-emerge (Table 1c, Figure 3). Therefore, flexible phenotypic plasticity and high photosynthetic capacity may contribute to the invasiveness of *A. philoxeroides* in both waterlogged and terrestrial habitats.

## Conclusions

Both invasive *A. philoxeroides* and native *A. sessilis* showed an escape strategy in response to waterlogging. However, *A. philoxeroides* showed less sensitivity of growth traits (lower plasticity), but higher waterlogging tolerance than *A. sessilis*. In addition, the two species differed in growth and physiological traits: *A. philoxeroides* displayed lower values of growth traits, but higher values of physiological traits. Higher tolerance of waterlogging and higher photosynthetic capacity may partly explain the ability of *A. philoxeroides* to invade waterlogged habitats.

## References

[1] Alpert P (2006) The advantages and disadvantages of being introduced. Biol Invasions 8: 1523–1534.

[2] Daehler CC (2003) Performance comparisons of co-occurring native and alien invasive plants: implications for conservation and restoration. Annu Rev Ecol Evol Syst 34: 183–211.

[3] He WM, Feng YL, Ridenour WM, Thelen GC, Pollock JL, et al (2009) Novel weapons and invasion: biogeographic differences in the competitive effects of *Centaurea maculosa* and its root exudate (+/−)-catechin. Oecologia 159: 803–815. 19219462 10.1007/s00442-008-1234-4

[4] Pyšek P, Jarošík V, Pergl J (2011) Alien plants introduced by different pathways differ in invasion success: unintentional introductions as a threat to natural areas. PLoS ONE 6: e24890. 21949778 10.1371/journal.pone.0024890 PMC3174229

[5] van Kleunen M, Dawson W, Schlaepfer D, Jeschke JM, Fischer M (2010) Are invaders different? A conceptual framework of comparative approaches for assessing determinants of invasiveness. Ecol Lett 13: 947–958. 20576028 10.1111/j.1461-0248.2010.01503.x

[6] Kolb A, Alpert P (2003) Effects of nitrogen and salinity on growth and competition between a native grass and an invasive congener. Biol Invasions 5: 229–238.

[7] Pyšek P, Křivánek M, Jarošík V (2009) Planting intensity, residence time, and species traits determine invasion success of alien woody species. Ecology 90: 2734–2744. 19886483 10.1890/08-0857.1

[8] Skálová H, Havlíčková V, Pyšek P (2012) Seedling traits, plasticity and local differentiation as strategies of invasive species of *Impatiens* in central Europe. Ann Bot 110: 1429–1438. 22247125 10.1093/aob/mcr316 PMC3489139

[9] Funk JL (2008) Differences in plasticity between invasive and native plants from a low resource environment. J Ecol 96: 1162–1173.

[10] Palacio-López K, Gianoli E (2011) Invasive plants do not display greater phenotypic plasticity than their native or non-invasive counterparts: a meta-analysis. Oikos 120: 1393–1401.

[11] Pyšek P, Richardson DM (2007) Traits associated with invasiveness in alien plants: where do we stand? Biol Invasions 193: 97–125.

[12] van Kleunen M, Weber E, Fischer M (2010) A meta-analysis of trait differences between invasive and non-invasive plant species. Ecol Lett 13: 235–245. 20002494 10.1111/j.1461-0248.2009.01418.x

[13] Radford IJ, Dickinson KJM, Lord JM (2007) Functional and performance comparisons of invasive *Hieracium lepidulum* and co-occurring species in New Zealand. Austral Ecol 32: 338–354.

[14] Willis AJ, Memmott J, Forrester RI (2000) Is there evidence for the post-invasion evolution of increased size among invasive plant species? Ecol Lett 3: 275–283.

[15] Godoy O, Valladares F, Castro-Díez P (2011) Multispecies comparison reveals that invasive and native plants differ in their traits but not in their plasticity. Funct Ecol 25: 1248–1259.

[16] Alpert P, Bone E, Holzapfel C (2000) Invasiveness, invasibility and the role of environmental stress in the spread of non-native plants. Perspect Plant Ecol Evol Syst 3: 52–66.

[17] Hawkes CV (2007) Are invaders moving targets? The generality and persistence of advantages in size, reproduction, and enemy release in invasive plant species with time since introduction. Am Nat 170: 832–843. 18171166 10.1086/522842

[18] van Kleunen M, Richardson DM (2007) Invasion biology and conservation biology: time to join forces to explore the links between species traits and extinction risk and invasiveness. Prog Phys Geog 31: 447–450.

[19] He WM, Li JJ, Peng PH (2012) A congeneric comparison shows that experimental warming enhances the growth of invasive *Eupatorium adenophorum* . PLoS ONE 7: e35681. 22536425 10.1371/journal.pone.0035681 PMC3334992

[20] Matzek V (2011) Superior performance and nutrient-use efficiency of invasive plants over non-invasive congeners in a resource-limited environment. Biol Invasions 13: 3005–3014.

[21] Xu C-Y, Schooler SS, Van Klinken RD (2012) Differential influence of clonal integration on morphological and growth responses to light in two invasive herbs. PLoS ONE 7: e35873. 22558248 10.1371/journal.pone.0035873 PMC3338812

[22] Colmer TD, Voesenek LACJ (2009) Flooding tolerance: suites of plant traits in variable environments. Funct Plant Biol 36: 665–681. 10.1071/FP09144 32688679

[23] Panda D, Sharma SG, Sarkar RK (2008) Chlorophyll fluorescence parameters, CO_2_ photosynthetic rate and regeneration capacity as a result of complete submergence and subsequent re-emergence in rice (*Oryza sativa* L.). Aquat Bot 88: 127–133.

[24] Bailey-Serres J, Voesenek L (2008) Flooding stress: Acclimations and genetic diversity. Annu Rev Plant Biol 59: 313–339. 18444902 10.1146/annurev.arplant.59.032607.092752

[25] Xiao Y, Tang JB, Qing H, Yan OY, Zhao YJ, et al (2009) Clonal integration enhances flood tolerance of *Spartina alterniflora* daughter ramets. Aquat Bot 92: 9–13.

[26] Dong BC, Liu RH, Zhang Q, Li HL, Zhang MX, et al (2011) Burial depth and stolon internode length independently affect survival of small clonal fragments. PLoS ONE 6: e23942. 21912652 10.1371/journal.pone.0023942 PMC3164666

[27] Xie D, Yu D (2010) Size-related auto-fragment production and carbohydrate storage in auto-fragment of *Myriophyllum spicatum* L. in response to sediment nutrient and plant density. Hydrobiologia 658: 221–231.

[28] Geng YP, Pan XY, Xu CY, Zhang WJ, Li B, et al (2006) Phenotypic plasticity of invasive *Alternanthera philoxeroides* in relation to different water availability, compared to its native congener. Acta Oecol 30: 380–385.

[29] Sun Y, Ding J, Frye MJ (2010) Effects of resource availability on tolerance of herbivory in the invasive *Alternanthera philoxeroides* and the native *Alternanthera sessilis* . Weed Res 50: 527–536.

[30] Maxwell K, Johnson GN (2000) Chlorophyll fluorescence-a practical guide. J Exp Bot 51: 659–668. 10938857 10.1093/jxb/51.345.659

[31] Wellburn R (1994) The spectral determination of chlorophylls *a* and *b*, as well as total carotenoids, using various solvents with spectrophotometers of different resolution. J Plant Physiol 144: 307–313.

[32] Morris DL (1948) Quantitative determination of carbohydrates with dreywood's anthrone reagent. Science 107: 254–255. 17814729 10.1126/science.107.2775.254

[33] Olano JM, Menges ES, Martinez E (2006) Carbohydrate storage in five resprouting Florida scrub plants across a fire chronosequence. New Phytol 170: 99–105. 16539607 10.1111/j.1469-8137.2005.01634.x

[34] Mahelka V (2006) Response to flooding intensity in *Elytrigia repens*, *E. intermedia* (*Poaceae*: *Triticeae*) and their hybrid. Weed Res 46: 82–90.

[35] Parolin P (2009) Submerged in darkness: adaptations to prolonged submergence by woody species of the Amazonian floodplains. Ann Bot 103: 359–376. 19001429 10.1093/aob/mcn216 PMC2707320

[36] van Eck W, Lenssen J, van de Steeg H, Blom C, de Kroon H (2006) Seasonal dependent effects of flooding on plant species survival and zonation: a comparative study of 10 terrestrial grassland species. Hydrobiologia 565: 59–69.

[37] Cosgrove DJ (2005) Growth of the plant cell wall. Nat Rev Mol Cell Bio 6: 850–861. 16261190 10.1038/nrm1746

[38] Sarkar R, Reddy J, Sharma S, Ismail AM (2006) Physiological basis of submergence tolerance in rice and implications for crop improvement. Curr Sci 91: 899–906.

[39] Mommer L, De Kroon H, Pierik R, Bögemann GM, Visser EJ (2005) A functional comparison of acclimation to shade and submergence in two terrestrial plant species. New Phytol 167: 197–206. 15948842 10.1111/j.1469-8137.2005.01404.x

[40] Yu L, Yu D, Liu C, Xie D (2010) Flooding effects on rapid responses of the invasive plant *Alternanthera philoxeroides* to defoliation. Flora 205: 449–453.

[41] Drenovsky RE, Khasanova A, James JJ (2012) Trait convergence and plasticity among native and invasive species in resource-poor environments. Am J Bot 99: 629–639. 22434772 10.3732/ajb.1100417

[42] Li H-L, Lei G-C, Zhi Y-B, An S-Q, Huang H-P, et al (2011) Nitrogen level changes the interactions between a native (*Scirpus triqueter*) and an exotic species (*Spartina anglica*) in coastal china. PLoS ONE 6: e25629. 21998676 10.1371/journal.pone.0025629 PMC3187781

[43] He WM, Thelen GC, Ridenour WM, Callaway RM (2010) Is there a risk to living large? Large size correlates with reduced growth when stressed for knapweed populations. Biol Invasions 12: 3591–3598.

[44] Kettenring KM, Mock KE (2012) Genetic diversity, reproductive mode, and dispersal differ between the cryptic invader, *Phragmites australis*, and its native conspecific. Biol Invasions 14: 2489–2504.

[45] Fan SF, Liu CH, Yu D, Xie D (2013) Differences in leaf nitrogen content, photosynthesis, and resource-use efficiency between *Eichhornia crassipes* and a native plant *Monochoria vaginalis* in response to altered sediment nutrient levels. Hydrobiologia 711: 129–137.

[46] Poorter H, Nagel O (2000) The role of biomass allocation in the growth response of plants to different levels of light, CO_2_, nutrients and water: a quantitative review. Aust J Plant Physiol 27: 595–607.

